# Effect of different walking break strategies on superficial femoral artery endothelial function

**DOI:** 10.14814/phy2.14190

**Published:** 2019-08-18

**Authors:** Sophie E. Carter, Richard Draijer, Sophie M. Holder, Louise Brown, Dick H. J. Thijssen, Nicola D. Hopkins

**Affiliations:** ^1^ Research Institute for Sport and Exercise Sciences Liverpool John Moores University Liverpool United Kingdom; ^2^ School of Sport York St John University York United Kingdom; ^3^ Unilever Research and Development Vlaardingen The Netherlands; ^4^ Unilever Research and Development Bedfordshire United Kingdom; ^5^ Department of Physiology Radboud Institute for Health Sciences Radboud University Medical Center Nijmegen The Netherlands

**Keywords:** Blood flow, flow‐mediated dilation, physical activity breaks, sedentary behavior, shear rate

## Abstract

Breaking up prolonged sitting with physical activity (PA) breaks prevents conduit artery dysfunction. However, the optimal break strategy to achieve this, in terms of the frequency or duration of PA, is not known. This study assessed the effect of breaking up sitting with different PA break strategies on lower limb peripheral artery endothelial function. Fifteen participants (10 male, 35.8 ± 10.2 years, BMI: 25.5 ± 3.2 kg m^−2^) completed, on separate days, three 4‐h conditions in a randomized order: (1) uninterrupted sitting (SIT), (2) sitting with 2‐min light‐intensity walking breaks every 30 min (2WALK), or (3) sitting with 8‐min light‐intensity walking breaks every 2 h (8WALK). At baseline and 4 h, superficial femoral artery function (flow‐mediated dilation; FMD), blood flow, and shear rate (SR) were assessed using Doppler ultrasound. For each condition, the change in outcome variables was calculated and data were statistically analyzed using a linear mixed model. There was no significant main effect for the change in FMD (*P* = 0.564). A significant main effect was observed for the change in blood flow (*P* = 0.022), with post hoc analysis revealing a greater reduction during SIT (−42.7 ± 14.2 mL·min) compared to 8WALK (0.45 ± 17.7 mL·min; *P* = 0.012). There were no significant main effects for mean, antegrade, or retrograde SR (*P* > 0.05). Superficial femoral artery blood flow, but not FMD, was reduced following uninterrupted sitting. This decline in blood flow was prevented with longer duration, less frequent walking breaks rather than shorter, more frequent breaks suggesting the dose (duration and frequency) of PA may influence the prevention of sitting‐induced decreases in blood flow.

## Introduction

Sedentary behavior is associated with increased risk of cardiovascular disease development and mortality independent of physical activity (PA) levels (Matthews et al. 2012; Biswas et al. 2015). Engagement in at least 1 h per day of moderate‐intensity PA can offset these adverse effects (Ekelund et al. 2016, 2019); however, such high levels of PA greatly exceed the current UK guidelines of 150 min of moderate‐intensity PA a week (Department of Health Physical Activity Health Improvement and Protection, 2011). Sitting is a highly prevalent sedentary behavior. Indeed, the UK office workers spend 60–65% of their work time sitting, which is not compensated with increased leisure‐time PA (Clemes et al. 2014; 2016). Importantly, sitting for up to 6 h leads to reductions in lower limb blood flow and shear stress, possibly due to decreased muscle activity and metabolic demand, resulting in acute conduit artery endothelial dysfunction, which is evident after only 1 h of sitting (Restaino et al. 2015; Thosar et al. 2014, 2015a,b; Walsh et al. 2017). Critically, endothelial dysfunction can contribute to the development of atherosclerosis (Deanfield et al. 2007; Lerman and Zeiher, 2005). These negative hemodynamic and vascular effects of sitting have, therefore, been suggested to be potential mechanisms for the increased cardiovascular disease incidence and mortality risk associated with sitting (Carter et al. 2017). Since arteries of the lower extremities are more prone to the development of atherosclerosis than the upper extremities (Sanada et al. 2005), and prolonged sitting has been shown to impair lower limb but not upper limb endothelial function (Thosar et al. 2014), interventions targeting lower limb endothelial function are needed.

The detrimental effect of sitting on endothelial function can be attenuated by breaking up sitting using short bouts of low‐intensity PA. Interrupting 3 h of sitting with three, 5‐minute walking bouts prevents a decrease in superficial femoral artery endothelial function, likely due to the repeated exposure of activity‐induced elevations in blood flow and shear rate (SR) (Thosar et al. 2015a). However, there is a dearth of other studies examining the influence of using PA breaks to interrupt sitting on endothelial function. Consequently, little is known regarding the optimal strategy or dose of activity needed to achieve this protective effect on the vasculature. Yet, the frequency of PA bouts may be a significant factor when breaking up sitting. Cross‐sectional studies have observed that individuals who accumulate their sedentary behavior in long, uninterrupted bouts have a worse cardiometabolic risk factor profile than individuals with the same total sedentary behavior, but who regularly interrupt this sedentary time with PA bouts (Healy et al. 2008; 2011). Consequently, the vascular effects of breaking up sitting with different frequencies of PA bouts need to be explored.

While frequent interruptions to sitting may be physiologically beneficial, practically this strategy may be difficult to employ in everyday scenarios such as the workplace. Alternatively, longer duration but less frequent PA breaks could represent a more feasible approach. Consequently, understanding the vascular responses to different PA break strategies has important practical significance for the development and implementation of guidelines to reduce sitting time. However, to date, no experimental research has examined the dose (duration and frequency) of PA break required to attenuate sitting‐induced superficial femoral artery endothelial dysfunction. The primary aim of this study was, therefore, to assess the effect of breaking up sitting with frequent, short‐duration PA breaks or longer, less frequent PA breaks on superficial femoral artery endothelial function. The superficial femoral artery was chosen to allow direct comparison with previous research utilizing a different break strategy that also assessed endothelial function in this artery (Thosar et al. 2015a). The secondary aim of this study was to assess the effect of each of these break strategies on blood flow and SR. It was hypothesized that a more frequent PA break protocol would increase blood flow and preserve endothelial function due to more regular elevations in blood flow and SR.

## Material and Methods

### Participants

Fifteen healthy desk workers (10 male) volunteered and written informed consent was obtained prior to inclusion. Participants were screened prior to testing for exclusion criteria including: age <20 and >60 years old, use of cardiovascular or metabolic medications, smoker, BMI > 35 or <18 kg∙m^−2^, use of hormone‐based contraception and diagnosis of cerebrovascular, cardiovascular, or metabolic disease. Study procedures were approved by the Liverpool John Moores University Ethics Committee and adhered to the Declaration of Helsinki.

### Study design

**Figure 1:**
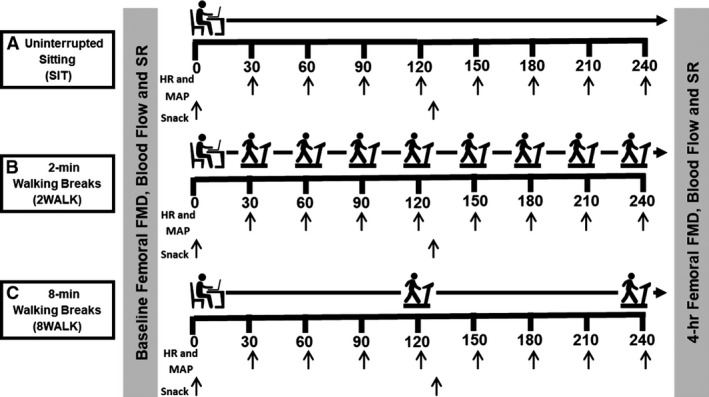
Experimental design for the three test conditions, completed in a randomized order, on three separate days. (A) 4‐h uninterrupted sitting (SIT), (B) Sitting with 2‐min treadmill walking breaks every 30 min (2WALK), (C) Sitting with 8‐min treadmill walking breaks every 120 min (8WALK). FMD, flow‐mediated dilation; SR, shear rate; HR, heart rate; MAP, mean arterial pressure

Participants attended the temperature‐controlled (20–22°C) laboratory at the same time of day (7.00–9.00 am) on three separate occasions. Testing procedures were the same across each test day (Fig. 1). After arrival and 20‐min supine rest, superficial femoral artery blood flow, SR, endothelial function (flow‐mediated dilation; FMD), mean arterial pressure (MAP), and heart rate (HR) were assessed in the supine position. Following baseline measurements, participants completed in a randomized order: (1) 4‐h uninterrupted sitting (SIT), (2) 4‐h sitting + 2‐min light‐intensity treadmill walking breaks every 30 min (2WALK), or (3) 4‐h sitting + 8‐min light‐intensity treadmill walking breaks every 120 min (8WALK). Participants were randomly assigned to the order in which they completed conditions using computer‐generated random numbers. Measurements were repeated immediately after the 4‐h condition. During each condition, MAP and HR were assessed every 30 min. HR was assessed immediately prior to and continuously during each walking break (Fig. 1).

### Study procedures

Prior to each visit, participants were instructed to avoid strenuous exercise for 24 h, to complete an overnight fast and abstinence from caffeine and alcohol. Women were assessed in the follicular phase of the menstrual cycle (days 1–7) in accordance with published guidelines for the assessment of FMD (Thijssen et al. 2011). All three visits occurred within a 7‐day period, firstly, this ensured that women were tested in the same phase of the menstrual cycle and secondly, this prevented participants changing their diet or PA levels which could otherwise influence our outcome measures. On their first visit, participants completed the International Physical Activity Questionnaire (Long form, IPAQ) (Booth, 2000) to determine habitual PA (Craig et al. 2003) and sitting time (Rosenberg et al. 2008). Given the duration of testing, on each visit, participants were given low calorie, low fat, low glycemic index, and standardized snacks at specified time points (Fig. 1). Following baseline tests, participants were given a breakfast cereal bar (Belvita Milk and Cereal Breakfast Biscuits, 220 kcal, 33.6 g carbohydrate, 7.2 g fat, 3.6 g protein) and a banana after 2 h (~100 kcal, ~27.0 g carbohydrate, ~0.3 g fat, ~1.0 g protein). Water was available to drink ad libitum.

### Conditions

#### Uninterrupted sitting (SIT)

Participants remained seated at a desk for 4 h and were permitted to perform desk‐based activities, for example reading, working on a computer. Participants were prevented from standing or walking, with the exception of visiting the toilet (walking distance of ~7.5 m; on average participants visited the toilet once during each condition). Owing to the potential acute effects of walking to the toilet on our outcome measures, participants were instructed to refrain from visiting the toilet in the last 1 h of the sitting period. Participants were supervised at all times to ensure that these conditions were adhered to. To create an ecologically valid sitting period, lower limb leg movement was uncontrolled.

#### 2‐min walking breaks (2WALK)

Sitting was interrupted every 30 min with a 2‐min light‐intensity treadmill walking break. Consequently, eight breaks were completed, totaling 16 min of activity. This break frequency was selected based on recommendations from The Sedentary Behaviour and Obesity Expert Working Group (Biddle, 2010) which advises taking a break from sitting every 30 min, while the duration of each walking break was based on previous research showing 2‐min walking breaks every 20 min lowers postprandial glucose and insulin concentrations (Dunstan et al. 2012). Walking was performed on a treadmill with no gradient (Run XT, Technogym, Italy) at a self‐selected, habitual walking speed to represent an ecologically valid PA break that could be performed in a working environment. Walking speed was determined during a familiarization session before the first experimental trial began and this speed was kept consistent for all walking breaks. Mean treadmill speed for each walk was 3.6 ± 0.9 km/h. Walking intensity was assessed during each PA bout using the Borg Rating of Perceived Exertion (RPE) scale (Borg, 1998) and HR.

#### 8‐min walking breaks (8WALK)

Sitting was interrupted every 120 min with an 8‐min light‐intensity walk, using the same walking speed as previously described. Consequently, two breaks were completed, totaling 16 min of activity. Therefore, the total duration of PA performed in both walking break conditions was identical. This less frequent break strategy was based on recommendations that interventions to break up sitting must be feasible (Benatti and Ried‐Larsen, 2015), which a high frequency breaks strategy may not be when translated into practise.

### Superficial femoral artery blood flow and endothelial function

Superficial femoral artery endothelial function was assessed since arteries of the lower extremities are more prone to atherosclerosis than those of the upper extremities (Sanada et al. 2005). The assessment of superficial femoral artery endothelial function was performed using the noninvasive FMD technique, according to the published guidelines (Thijssen et al. 2011). A rapid inflation and deflation pneumatic cuff (D.E. Hokanson, Bellevue, WA) was positioned around the right thigh, above the patella. To image the right superficial femoral artery, a 10‐MHz multi‐frequency linear array probe attached to high resolution ultrasound machine (T3000; Terason, Burlington, MA) was used. Images were acquired above the occlusion cuff and distal from the artery bifurcation. For the acquisition of arterial diameters, ultrasound parameters were adjusted to optimize the B‐mode image of the lumen‐arterial wall interface. Once a satisfactory image was obtained, the probe was held consistently in this position. Arterial blood flow was simultaneously assessed via Doppler ultrasound using the same ultrasound machine with a consistent insonation angle of 60° for each assessment. Baseline arterial diameter and blood flow were recorded for 1 min. Following this, the cuff was inflated to 220 mmHg for 5 min to induce local ischemia. After cuff deflation, arterial diameter and blood flow recordings were continued for a further 3 min. This procedure was repeated for each endothelial function assessment and all measures were completed by the same sonographer. The sonographer had a between‐day coefficient of variation of 11.7% for superficial femoral artery FMD.

Data analysis was performed using custom‐designed automatic edge‐detection and wall‐tracking software, a reproducible and valid method (Woodman et al. 2001; Green et al. 2002) which is largely independent of investigator bias (Woodman et al. 2001). The software enables regions of interest (ROI) to be selected from the initial frame of each data file for the B‐mode image and Doppler waveform. The analysis process has been described in detail elsewhere (Black et al. 2008), but briefly for diameter analysis, the ROI was selected based on the clarity of the B‐mode image and the distinction between the arterial wall‐lumen interface. A second ROI was selected to encompass the Doppler waveform, which then automatically detects the peak of the waveform. Each frame was subsequently analyzed at a rate of ~30 Hz, enabling synchronized arterial diameter, blood velocity, blood flow (arterial cross‐sectional area x blood velocity), and SR data to be acquired. SR (4 × [blood velocity/arterial diameter]) was used as an estimation of shear stress due to the inability to measure blood viscosity.

For the FMD assessment, baseline arterial diameter was determined as the mean of the data acquired 1 min prior to cuff inflation. Following cuff deflation, peak vessel diameter was automatically calculated using the custom‐designed software. This process is described in detail elsewhere (Black et al. 2008), but briefly involves an algorithm which identifies the maximum bracket of data using a moving‐window smoothing function. The maximum value from this process is determined as peak vessel diameter. From this data, superficial femoral artery FMD (%) was calculated as the percentage change in vessel diameter from baseline diameter (([peak arterial diameter‐baseline arterial diameter]/baseline arterial diameter) × 100 %). SR area under the curve (AUC) was calculated from post cuff deflation until the point of peak vessel diameter, using the automated software and formulated using the trapezoid rule. Blood flow and SR data were analyzed from the 1‐min baseline period during the FMD test. Mean blood flow and SR were determined as an average of this 1‐min period. The patterns of SR were also assessed by calculating the AUC for antegrade blood flow and SR and the retrograde blood flow and SR recordings, as described in detail elsewhere (Green et al. 2002). Vascular conductance was calculated by dividing mean blood flow by MAP.

### Hemodynamics

MAP and HR were measured with an oscillometric cuff at the left brachial artery (Carescape V100, Dinamap, GE Healthcare, UK) at baseline, 4 h, and every 30 min during each condition. During each walking break, HR was continuously assessed using a HR monitor (Polar FT1 Heart Rate Monitor, Polar Electro, Finland) and averaged over the respective walking break duration.

### Statistical analyses

Data were analyzed using statistical software (SPSS Version 22.0, IBM Corporation, Somers, NY), with significance accepted as *P* ≤ 0.05. Results are presented as means ± standard deviation (SD). SD was calculated from the adjusted standard error (SE) derived from statistical analyses (SD = SE × √N). For each condition, the change in all outcome parameters (FMD, arterial diameter, SR, blood flow, MAP, HR) was calculated (4‐h – baseline, Δ). These parameters were subsequently analyzed using one‐factor general linear mixed model with participants specified as a random factor, condition specified as a fixed factor, and baseline values as a covariate to assess differences between each condition. FMD was also analyzed using an allometric approach that controls for changes in baseline diameter (Atkinson and Batterham, 2013). In accordance with the published guidelines (Atkinson et al. 2009), the relationship between FMD and SR was examined to determine if ratio normalization should be applied. General linear mixed models were used to assess the effect of “condition” and “time” on MAP and HR measured every 30 min during each condition. Differences in HR between pre‐walk and during each walk were analyzed using paired samples *t*‐tests. Post hoc analyses were performed using the least significant difference (LSD) method. Effect size (Cohen's *d*) of all significant differences was calculated by dividing the difference in group means by the standard deviation of the pooled data. These were interpreted as: *d* = 0.2 considered small, *d* = 0.5 considered medium, and *d* = 0.8 considered large (Cohen, 1988). Sample size was based on previous work showing significant changes in superficial femoral artery endothelial function following uninterrupted sitting and interrupting sitting with PA breaks using a sample of 12 healthy adults (Thosar et al. 2015a). Thus, adequate statistical power was aided using a sample of 15 healthy adults in a within‐subject study design.

## Results

Participants had a mean age of 35.8 ± 10.2 years, a mean body mass of 74.5 ± 11.9 kg, height of 170.8 ± 8.9 cm, and a BMI of 25.5 ± 3.2 kg∙m^−2^. Eight, five, and two participants were classified as healthy weight, overweight, and obese, respectively. Resting HR was 59 ± 3 bpm and MAP was 83 ± 10 mmHg. Participants self‐reported spending 8.2 ± 2.2 h (range: 6–12 h) sitting during weekdays and 6.0 ± 1.9 h (range: 3–10 h) sitting during weekends, totaling 53.2 ± 12.4 h of sitting per week. Self‐report PA was 4524.3 ± 2098.7 MET‐minutes/week (range: 2153–9852 MET‐minutes/week). Based on IPAQ scoring, 13 participants were classified as having high PA levels and two participants were classified as having moderate PA levels.

### Hemodynamic measures

There was no significant main effect for the change in MAP (*P* = 0.778) or the change in HR (*P* = 0.895; Table 1).

**Table 1:** For each condition, heart rate (HR), mean arterial pressure (MAP), superficial femoral artery flow‐mediated dilation (FMD), blood flow, and shear rate (SR) at baseline, 4 h, and the overall change (*∆*) with statistically adjusted baseline covariate control (*n* = 15). (Values are mean ± SD [95% confidence interval])

	SIT			2WALK			8WALK			*P*‐value
	Baseline	4 h	∆#	Baseline	4 h	∆#	Baseline	4 h	∆#	
HR (bpm)	59 ± 12	56 ± 8	−2 ± 6 [−5, 1]	58 ± 9	55 ± 12	−3 ± 10 [−9, 3]	56 ± 8	55 ± 7	−2 ± 7 [−6, 2]	0.895
MAP (mmHg)	83 ± 10	84 ± 9	2 ± 7 [−2, 6]	80 ± 7	84 ± 8	3 ± 6 [−1, 7]	81 ± 8	83 ± 10	2 ± 8 [−3, 7]	0.778
FMD (%)	5.5 ± 3.1	6.9 ± 4.0	0.9 ± 3.7 [−1.3, 3.1]	6.7 ± 3.4	7.4 ± 3.1	1.0 ± 3.2 [−0.9, 2.8]	6.7 ± 3.8	8.9 ± 4.6	2.4 ± 4.6 [−0.3, 5.2]	0.564
FMD (%) allometric modelling	5.8 ± 2.6	6.9 ± 3.3	0.7 ± 3.9 [−1.7, 3.0]	6.9 ± 2.6	7.4 ± 2.8	1.0 ± 3.1 [−0.8, 2.9]	6.7 ± 3.2	8.0 ± 3.4	2.5 ± 4.2 [−0.1, 5.1]	0.491
SR AUC (s^−1^ × 10^3^)	16.9 ± 11.4	16.9 ± 10.4	−0.7 ± 9.9 [−6.7, 5.3]	15.6 ± 10.1	18.5 ± 10.2	1.1 ± 10.8 [−5.4, 7.7]	20.9 ± 10.2	29.3 ± 20.7	10.9 ± 19.5 [−1.0, 22.8]	0.076
Baseline diameter (cm)	0.62 ± 0.11	0.61 ± 0.11	−0.02 ± 0.04 [−0.03, −0.00]	0.62 ± 0.10	0.62 ± 0.11	−0.00 ± 0.04 [−0.02, 0.02]	0.63 ± 0.08	0.61 ± 0.12	−0.02 ± 0.07 [−0.06, 0.01]	0.255
Mean blood flow (mL·min)	182.4 ± 66.7	151.8 ± 56.4	−42.7 ± 53.1* [−73.8, −11.6]	213.8 ± 109.7	178.1 ± 91.0	−19.8 ± 91.7 [−72.7, 33.2]	191.6 ± 81.5	195.9 ± 65.5	0.45 ± 66.2 [−37.9, 38.8]	0.022
Vascular conductance (mL·min·mmHg)	2.2 ± 0.8	1.8 ± 0.7	−0.6 ± 0.7* [−0.9, −0.2]	2.6 ± 1.2	2.2 ± 1.1	−0.3 ± 1.1 [−0.9, 0.4]	2.3 ± 1.0	2.3 ± 0.8	−0.1 ± 0.9 [−0.6, 0.4]	0.046
Mean SR (s^−1^)	61.6 ± 25.3	57.5 ± 28.3	−4.4 ± 16.5 [−14.0, 5.2]	65.3 ± 21.5	66.8 ± 55.2	1.9 ± 48.6 [−26.2, 30.0]	63.9 ± 34.8	78.2 ± 61.8	14.4 ± 64.0 [−22.6, 51.4]	0.390
AntegradeSR (s^−1^)	82.1 ± 27.4	76.2 ± 27.6	−5.8 ± 16.5 [−15.3, 3.8]	83.4 ± 24.6	82.0 ± 52.9	−0.9 ± 44.5 [−26.6, 24.8]	79.9 ± 35.9	91.6 ± 59.6	11.2 ± 60.2 [−23.6, 45.9]	0.402
RetrogradeSR (s^−1^)	−20.5 ± 11.2	−18.7 ± 8.3	0.9 ± 4.5 [−1.7, 3.5]	−18.1 ± 7.9	−15.2 ± 11.1	3.0 ± 8.6 [−2.1, 8.0]	−16.0 ± 7.3	−13.4 ± 9.3	3.5 ± 7.9 [−1.1, 8.0]	0.464

SIT, uninterrupted sitting; 2WALK, 2‐min walking breaks; 8WALK, 8‐min walking breaks; HR, heart rate; MAP, mean arterial pressure; FMD, flow‐mediated dilation; SR, shear rate, AUC, area under the curve.

^#^ Delta change values expressed with statistically adjusted baseline covariate control.

* Significantly different to 8WALK (*P* < 0.05).

### Superficial femoral artery blood flow and endothelial function

**Figure 2:**
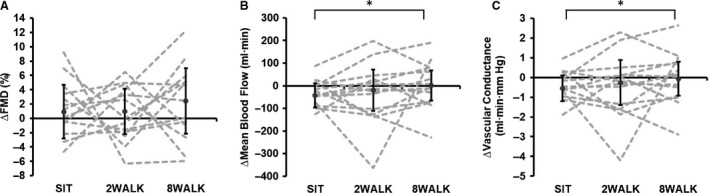
Change in superficial femoral artery (A) flow‐mediated dilation (FMD), (B) mean blood flow, and (C) vascular conductance measured at baseline and after 4 h of each experimental condition, with statistically adjusted baseline covariate control. SIT‐ uninterrupted sitting; 2WALK‐ 2‐min walking breaks; 8WALK‐ 8‐min walking breaks. Error bars = ±SD. ^*^Significant difference between conditions (*P* < 0.05)

**Figure 3:**
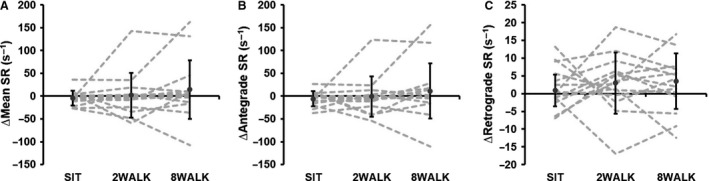
Change in superficial femoral artery (A) mean, (B) antegrade, and (C) retrograde shear rate (SR) measured at baseline and after 4 h of each experimental condition, with statistically adjusted baseline covariate control. SIT‐ uninterrupted sitting; 2WALK‐ 2‐min walking breaks; 8WALK‐ 8‐min walking breaks. Error bars = ±SD

Values for superficial femoral artery blood flow and FMD are presented in Table 1. No significant main effect was observed for the change in superficial femoral artery FMD when data were analyzed using general linear mixed model (*P* = 0.564; Fig. 2) or using allometric modeling analysis (*P* = 0.842; Table 1). There was also no significant main effect for SR AUC (*P* = 0.076; Table 1). The change in superficial femoral FMD was not correlated to the change in SR AUC (*r* = 0.078, *P* = 0.638), therefore in line with the published guidelines (Atkinson et al. 2009), FMD was not normalized to the SR stimulus. Peripheral blood flow data are shown in Table 1. A significant main effect was observed for the change in superficial femoral artery blood flow (*P* = 0.022; Fig. 2), with post hoc analysis revealing a greater reduction in blood flow during SIT compared to 8WALK (*P* = 0.012, *d* = 0.64), but not between SIT and 2WALK (*P* = 0.393, *d* = 0.32). A significant main effect was also observed for the change in superficial femoral artery vascular conductance (*P* = 0.046; Fig. 2), with post hoc analysis revealing a greater reduction in blood flow during SIT compared to 8WALK (*P* = 0.020, *d* = 0.90), but not between SIT and 2WALK (*P* = 0.339, *d* = 0.34). There was no significant main effect observed for mean (*P* = 0.390), antegrade (*P* = 0.402), or retrograde (*P* = 0.464) SR (Fig. 3).

### Sitting periods

HR and MAP taken every 30 min during each condition are shown in Table 2. For HR, there was a significant effect for time (*P* < 0.001). Post hoc analyses showed that compared to 30 min, HR was significantly reduced at all other time points (*P* < 0.001) except at 1 h in the sitting period (*P* = 0.274). No significant condition (*P* = 0.261) or interaction effects (*P* = 0.598) were observed. For MAP, no significant time (*P* = 0.965), condition (*P* = 0.447), or interaction (*P* = 0.809) effects were observed.

**Table 2:** Heart rate (HR) and mean arterial pressure (MAP) every 30 min for each 4‐h condition (*n* = 15). (Values are mean ± SD)

	Time							
	30 min	1 h	1 h 30 min*	2 h*	2 h 30 min*	3 h*	3 h 30 min*	4 h*
HR (bpm)								
SIT	64 ± 11	62 ± 9	61 ± 10	58 ± 10	60 ± 9	61 ± 10	61 ± 11	58 ± 10
2WALK	62 ± 10	64 ± 12	61 ± 9	61 ± 10	58 ± 9	59 ± 10	59 ± 10	58 ± 12
8WALK	65 ± 11	62 ± 12	61 ± 10	58 ± 10	57 ± 9	58 ± 10	59 ± 10	59 ± 13
MAP (mmHg)								
SIT	87 ± 8	86 ± 9	86 ± 9	85 ± 8	89 ± 10	85 ± 7	86 ± 9	85 ± 7
2WALK	87 ± 14	87 ± 14	86 ± 12	88 ± 9	86 ± 12	86 ± 12	87 ± 10	88 ± 9
8WALK	88 ± 12	86 ± 10	86 ± 10	86 ± 11	87 ± 8	87 ± 9	88 ± 12	88 ± 10

SIT, uninterrupted sitting; 2WALK, 2‐min walking breaks; 8WALK, 8‐min walking breaks; HR, heart rate; MAP, mean arterial pressure.

* Significant main effect for time (*P* < 0.001). Indicates significantly reduced compared to 30 min (*P* < 0.001).

### Walking breaks

For 2WALK and 8WALK, mean treadmill speed for each condition and every walking break was 3.6 ± 0.9 km/h (range: 2–5 km/h) at an RPE of 8.6 ± 0.9 (range: 7–10). In 2WALK, HR significantly increased during each walking break, with an average increase of 33 ± 1 bpm (Pre‐Walk: 61 ± 2 bpm; Walking: 94 ± 2 bpm, *P* < 0.001). During 8WALK, both walking breaks significantly increased HR, with an average increase of 37 ± 2 bpm (Pre‐Walk: 69 ± 3 bpm; Walking: 96 ± 6 bpm, *P* < 0.001).

## Discussion

This study demonstrates, for the first time, that the dose (duration and frequency) of PA used to interrupt sitting influences the peripheral artery blood flow. Reductions in superficial femoral artery blood flow following 4 h of prolonged, uninterrupted sitting were prevented with less frequent, longer duration walking breaks (8WALK) but not more frequent, shorter duration waking breaks (2WALK). Neither sitting nor PA breaks significantly altered superficial femoral artery endothelial function or affected mean arterial SR or SR patterns. Our results suggest that longer duration walking breaks may be more effective than shorter, more frequent breaks in preventing the decline in lower limb blood flow during prolonged sitting.

This study is the first to compare the effects of different PA break strategies to interrupt sitting on peripheral vascular function. Breaking up sitting with 5‐minute walking breaks prevents superficial femoral artery endothelial dysfunction (Thosar et al. 2015a); however, contrary to this research, our findings indicate no difference in endothelial function between prolonged sitting and the 2WALK or 8WALK conditions. This difference is likely due to the permission of habitual, unstandardized lower limb leg movement during sitting in our study, while Thosar et al. restricted all limb motion. Such small leg movements in our sitting condition may have maintained endothelial function and contributed to the small increase that was observed. Indeed, low level muscular contractions while sitting for 3 h via leg fidgeting for 1 minute every 4 minutes maintained popliteal artery blood flow, in turn preventing the decrease in endothelial function that otherwise occurred (Morishima et al. 2016). Interestingly, and in contrast to endothelial function, in our study prolonged sitting caused a decline in superficial femoral artery blood flow despite this permissive leg movement. Importantly, chronically decreased limb perfusion can contribute to heightened cardiovascular disease risk (Dinenno et al. 2001; Anton et al. 2006). However, previous studies that have observed sitting‐induced superficial femoral artery endothelial dysfunction do not report blood flow data (Thosar et al. 2014; Thosar et al. 2015a,b); thus, the magnitude of the reduction in blood flow and therefore shear stress needed to induce dysfunction is unknown. It is possible that in healthy adults, larger reductions in blood flow than observed in this study are needed to mediate a decline in superficial femoral artery endothelial function. Instead, long‐term repeated exposure to sitting‐induced decreases in blood flow and shear stress may contribute endothelial dysfunction. Indeed, chronically reduced blood flow and shear stress in turn downregulates nitric oxide production leading to endothelial dysfunction, creating a proatherogenic environment (Johnson et al. 2011). Further research should therefore explore the long‐term influence of prolonged sitting on lower limb blood flow and endothelial function.

The mechanisms underlying the observed sitting‐induced decline in superficial femoral artery blood flow may relate to hemodynamic and metabolic factors. While sitting, the decline in skeletal muscle activity in the lower limbs, thus decreased energy demand, may have lowered blood flow to this region. Indeed, muscle activity in the hamstrings and quadricep muscles is lower while sitting compared to standing and walking (Pesola et al. 2015). Furthermore, the absence of muscle contractions may lead to blood pooling in the lower limbs (Morishima et al. 2016). In support, 3 h of uninterrupted sitting increases ankle (Morishima et al. 2016) and calf (Restaino et al. 2015; Vranish et al. 2017) circumferences, indicating venous pooling. An acute period of uninterrupted sitting also leads to elevated plasma fibrinogen and reduced plasma volume, increasing blood viscosity (Howard et al. 2013). It is therefore plausible that a similar response may have occurred in our study, leading to a reduction in blood flow. Further research is required to explore these potential mechanisms.

The decline in superficial femoral artery blood flow was prevented in the 8WALK condition and not the 2WALK condition, suggesting the duration of PA may be more important than the frequency of the PA breaks for preserving blood flow during periods of prolonged sitting. In support, previous work found that superficial femoral artery endothelial function was maintained using 5‐min walking breaks, completed at 30‐, 90‐, and 150 min during 3 h of sitting (Thosar et al. 2015a), therefore also adopting an infrequent break strategy. Taken together, these data suggest at least a 5‐ to 8‐minute PA break may be needed every 1–2 h to prevent sitting‐induced impairments in peripheral blood flow and endothelial function. Further research is needed to determine the minimum required PA duration and break frequency to effectively maintain peripheral blood flow and function during sitting. The maintenance of peripheral blood flow during longer walking breaks in our study is perhaps due to the more sustained activation of the lower limb muscle mass during walking which would stimulate activity‐induced increases in blood flow (Joyner and Casey, 2015). Indeed, muscle blood flow is closely matched to the metabolic demands of contraction (Joyner and Casey, 2015), therefore the longer duration walking breaks in the 8WALK condition would have likely led to a heightened blood flow response due to more muscle contractions and an increased metabolic demand compared to the 2WALK condition. It is possible that the longer duration PA in the 8WALK condition may have caused the production of local vasodilators, such as nitric oxide, in skeletal muscle which could have prevented peripheral arteriole vasoconstriction, maintaining higher peripheral blood flow (Joyner and Casey, 2015). Further research examining these potential mechanisms is needed.

Mean, antegrade, and retrograde SR did not differ between the three conditions. This supports the previous work showing no difference in mean SR between prolonged sitting for 3 h and a walking break intervention to interrupt this sitting time (Thosar et al. 2015a). However, in this study and in our work, measures of SR were taken over 20 minutes following the PA breaks, therefore any increases in SR may have returned to baseline at the 4‐hr measurement time point. Indeed, in the brachial artery, breaking up sitting with callisthenics caused transient increases in mean SR which returned to baseline levels 20 minutes following the final PA break (Carter and Gladwell, 2017). Future research is therefore needed to profile the immediate SR responses in the lower limbs to PA breaks as this would heighten the mechanistic understanding of the effects of breaking up sitting with PA bouts.

Interventions to break up sitting must be feasible (Benatti and Ried‐Larsen, 2015), otherwise any potential health benefits from reducing sitting time will not be translated to target population groups such as highly sedentary office workers. This study therefore compared two different approaches to incorporating the same volume of PA into a prolonged sitting period, by manipulating the frequency and duration of the PA breaks. As discussed, longer duration but less frequent walking breaks compared to shorter duration, more frequent breaks attenuated the reduction in lower limb peripheral artery blood flow associated with prolonged sitting. A high frequency breaks strategy may not be suitable in certain sedentary workplaces such as contact or call center roles where time at the desk is monitored and specific break periods are allocated by employers. In such cases, employees should be encouraged to use this break opportunity to take a walking break rather than remaining sedentary. Furthermore, frequently breaking up sitting may be too disruptive to certain job tasks, such as reading documents and answering the phone. In such cases, a longer, less frequent break protocol, such as that proposed in this study, could be suitable. Since this study was conducted in a laboratory environment, future research should assess the feasibility of the 8WALK strategy in ecologically relevant environments, such as the workplace.

Despite the potential practical benefits of adopting a low frequency PA break strategy in the workplace, this recommendation contradicts the existing research examining the effects of breaking up sitting on metabolic health markers (Healy et al. 2008; Peddie et al. 2013) and blood flow to other vascular beds (Carter et al. 2018). Indeed, a more frequent break protocol is more effective at reducing postprandial glucose and insulin concentrations (Peddie et al. 2013) and maintaining cerebral blood flow (Carter et al. 2018). Such studies have employed PA breaks every 20‐ or 30 min during sitting, whereas we observed benefits to peripheral blood flow taking a PA break every 2 h. It may therefore be that a break frequency between these two time points can prevent sitting‐induced impairments in both vascular and metabolic health. Future research should therefore examine the effects of different PA break strategies on both vascular and metabolic health outcomes to develop an optimal break frequency and duration for overall cardiometabolic health.

### Limitations

Our study assessed the responses to an acute sedentary period, therefore the chronic impact of uninterrupted sitting or PA breaks on vascular function is not known. The study only included healthy, sedentary workers, therefore larger responses to prolonged sitting may have been observed in clinical populations or those from less sedentary occupations. Indeed, in less sedentary workers, the enforced sedentary time would be a greater change to their habitual activity levels. In contrast, the desk‐based participants who were recruited for this study may already exhibit some sitting‐induced endothelial dysfunction if they have been exposed to chronic prolonged sitting periods prior to the study (e.g. working in an office for several years). As discussed, leg movement in the prolonged sitting condition was uncontrolled which may have attenuated any impairment in endothelial function; however, permissive lower limb movement was deemed to be ecologically representative of habitual sitting behavior. Furthermore, we did not quantify leg movement during sitting, which could provide evidence to support the mechanistic explanation for a lack of impairment in superficial femoral artery FMD in our data. Whether similar vascular responses would occur using different PA modalities to break up sitting, such as standing and simple resistance exercises, are not known and should be explored in future research. Measures of lower limb blood pooling, such as calf circumference, were not included, which may have explained the observed reduction in blood flow. Due to a disproportionate representation of males and females, we were not able to control for sex in our analyses, which may have influenced the vascular responses to sitting (Vranish et al. 2017). Finally, we did not include any blood markers, for instance, vasodilators such as nitric oxide, which could have provided mechanistic insight into the differing effects of the PA break strategies on superficial femoral artery blood flow.

## Conclusion

While breaking up sitting with two different PA break strategies had no influence on superficial femoral artery endothelial function, this study demonstrates that the type of break strategy used can influence vascular blood flow responses. Reductions in lower limb peripheral artery blood flow associated with prolonged sitting are attenuated using longer duration but less frequent walking breaks compared to shorter duration, more frequent breaks. Collectively, these findings provide an insight into the dose, in terms of frequency and duration, of PA needed to prevent sitting‐induced blood flow reductions, which could have important implications for long‐term endothelial function, and further research should explore this possibility. Further research is also required to better understand the importance of different PA break durations and frequencies to develop the optimal dose of PA to break up prolonged sitting.

## Conflict of Interest

SEC received PhD scholarship funding from a Biotechnology and Biological Sciences Research Council (BBSRC) grant. RD and LB are employed by Unilever, which has commercial interests in Food, Home and Personal Care products. All other authors declare they have no conflict of interest.
